# Long-Term Consequences of Puumala Hantavirus Infection

**DOI:** 10.3390/v14030598

**Published:** 2022-03-14

**Authors:** Jukka Mustonen, Antti Vaheri, Ilkka Pörsti, Satu Mäkelä

**Affiliations:** 1 Faculty of Medicine and Health Technology, Tampere University, 33014 Tampere, Finland; ilkka.porsti@tuni.fi; 2 Department of Internal Medicine, Tampere University Hospital, 33520 Tampere, Finland; satu.m.makela@pshp.fi; 3 Department of Virology, Medicum, University of Helsinki, 00290 Helsinki, Finland; antti.vaheri@helsinki.fi

**Keywords:** Puumala hantavirus, hemorrhagic fever with renal syndrome, postinfectious glomerulonephritis

## Abstract

Several viral infections are associated with acute and long-term complications. During the past two years, there have been many reports on post-infectious symptoms of the patients suffering from COVID-19 disease. Serious complications occasionally occur during the acute phase of Puumala orthohantavirus caused nephropathia epidemica. Severe long-term consequences are rare. Fatigue for several weeks is quite common. Hormonal insufficiencies should be excluded if the patient does not recover normally.

## 1. Introduction

Nephropathia epidemica (NE), an acute infectious disease caused by Puumala orthohantavirus (PUUV) distributed by the bank vole (*Myodes glareolus*), is clinically characterized by high fever, thrombocytopenia, acute kidney injury (AKI) and increased capillary permeability [1,2]. The infection is usually mild or moderate in severity, but some patients need treatment in an intensive care unit. The mortality rate is very low ranging from 0.08% to 0.4% [2]. Irreversible shock or multiple bleedings have been the most common causes of death [1,2]. About 2500 serologically confirmed cases are reported annually in Finland, but from the seroprevalence studies, it can be calculated that only 20–30% of infected humans seek medical attention leading to serological diagnosis [2]. PUUV infections are quite common also in Northern Sweden, European Russia and in Germany [1,2].

Several severe complications have been described in the acute phase of PUUV-caused hemorrhagic fever with renal syndrome (HFRS) [2]. Many of them can lead to a long hospital treatment and even to a lethal outcome (Table 1). Most patients recover totally from the disease. Long-term consequences, however, can occur. Cardiovascular, nephrological, endocrinological, and some other consequences have been reported (Table 2). 

## 2. Cardiovascular System

A Swedish study has shown that there is a significantly increased risk for acute myocardial infarction (AMI) and stroke in the immediate period after PUUV caused HFRS [3]. The time periods of 21 and 90 days after HFRS were compared with a control period ending at 365 days after the infection. The incidence rate ratios for AMI were 5.53 to 6.02 and those for stroke were 12.93 to 15.16, respectively. Neither sex nor age of the patients interacted with the associations observed in the study [3]. According to the same study group, cardiovascular diseases might be overrepresented as a cause of death in the year after the acute phase of the disease [4]. We have unpublished results that PUUV infection is a risk factor for AMI also in Finnish males aged over 50 years. 

An increased risk for venous thromboembolism (VTE) during the first two weeks following HFRS onset has been reported from Sweden [5]. During this time period, the risk for deep vein thrombosis in comparison with control periods was 45.9 (95% CI, 18–117.1). Females were observed to have a higher risk for VTE compared with males [5]. Many kinds of infections are known to be associated with increased risk for venous thrombosis [6]. There are many well-known interactions between hantavirus infection, inflammatory mediators, the endothelium, the complement system, and the coagulation system, which can be associated with this complication [7]. 

The results of several studies performed in many countries have suggested that various hantavirus infections may predispose some patients to the development of hypertension [2]. In Europe, reports among patients with PUUV and Dobrava virus infections have come from Sweden, France, Croatia, and Bosnia and Herzegovina [2]. This was also the conclusion in our previous follow-up studies in Finland with a limited number of patients with PUUV infection [8,9]. We have also reported that some patients have increased amount of tubular proteinuria six years after the acute phase of NE [10]. It is possible that this may be a marker of a slight chronic renal damage that may have an influence on the development of hypertension in some individuals. 

## 3. Lymphoma

Swedish scientists have followed about 6600 individuals diagnosed with PUUV-associated HFRS in Sweden from 1997 to 2011, the mean follow-up time being 6.4 years. They reported a 73% increased risk for lymphoma [11]. The number of observed cases, however, was rather low as lymphoma was observed in 26 HRFS-diagnosed individuals in comparison to 15 cases among the control population. The risk of other forms of malignancies did not differ significantly between the study groups [11]. 

An increase in lymphoma frequency is interesting as PUUV is known to infect lymphoid tissue including the spleen. By using magnetic resonance imaging (MRI) it has been observed that during the acute phase of NE, spleen is enlarged in all patients [12]. It is also known that a strong and long-lasting lymphoid cell response takes place during hantavirus infections [1]. 

## 4. Renal Complications

A typical finding in NE is AKI which, in most cases, is mild or moderate in severity [13]. Transient hemodialysis treatment is needed in about five percent of the hospital-treated patients. Renal ultrasound shows transiently enlarged kidneys in most patients [14] and morphologically the renal disease is acute hemorrhagic tubulointerstitial nephritis [13]. 

The prognosis of AKI is favorable and renal function as measured by plasma creatinine concentration, normalizes also in patients with severe AKI [15]. Albuminuria makes a flash-like appearance, and it returns to normal levels within 2–3 weeks [16]. In the case of AKI caused by various other etiologies, this is a quite unique phenomenon. There are some reports about slightly decreased renal tubular function and glomerular hyperfiltration observed some years after PUUV infection [2,13]. The real significance of these findings is not known. 

In rare cases, PUUV infection can cause so called post-infectious glomerulonephritis [17]. Clinically, it manifests as the nephrotic syndrome two to three weeks after NE, i.e., at the convalescent phase of the disease. As a new symptom, the patient notices peripheral swellings in the legs, while nephrotic amount of proteinuria (>3.5 g/day) as well as hypoalbuminemia are found in the laboratory examinations. Twelve such cases have been reported in Finland [18,19]. Renal biopsy finding in most cases was membranoproliferative glomerulonephritis (GN). This type of GN is known to be caused by many viral infections including hepatitis B and C. The prognosis of the patients was good, and the nephrotic syndrome resolved spontaneously or due to the immunosuppressive drug therapy the patients had received. Only one of the 12 patients progressed to chronic renal failure and needed chronic dialysis treatment [18,19]. 

## 5. Endocrinology 

When evaluated using MRI, the pituitary gland expands during acute NE, but later returns to the normal size [20]. Pituitary hemorrhage and hormonal insufficiency, i.e., hypopituitarism may complicate recovery from acute NE [2,20]. Hormonal deficiencies are common during the acute phase of the disease [21,22]. In a follow-up study performed in Finland with a median follow-up 5 years, 9/54 (17%) of patients were diagnosed with a chronic, overt hormonal deficit. Both hypopituitarism and primary hypothyroidism were found in 5/54 (9%) patients. In addition, chronic subclinical testicular failure was found in five (9%) men [21]. One patient developed autoimmune polyendocrinopathy and hypophysitis [23]. It is, however, not always clinically evident, if a hormonal deficiency that developed after NE was caused by PUUV infection or by some other etiology [20]. 

A review on hypopituitarism after orthohantavirus infections has recently been published [24]. According to the review, the pathogenesis of this rare complication remains unknown. Both ischemic and hemorrhagic damage of the pituitary gland are possible. Ischemic damage can be caused by hypotension and/or vasospasm during the acute phase of the disease. Most often the hormonal defects were detected from two months up to thirteen months post-infection [24]. This complication should be kept in mind by the clinician if the patient does not recovery normally from PUUV infection. Long-lasting fatigue is sometimes a clinical symptom of a developed hormonal deficiency (see below). 

Several kinds of central nervous system and ocular manifestations are common during acute PUUV infection [25]. It is quite unusual that other chronic sequences except the hormonal ones, develop afterwards in these patients [25]. 

## 6. Other Consequences 

Dr. Juhani Lähdevirta reported in 1971 in his academic thesis findings on clinically defined cases of NE [26]. He found that three weeks after the acute phase many patients suffered from various symptoms, the most common being backache, muscular weakness, fatigue, and headache. The mean duration of disability to perform normal work was one month, the range being from 15 to 48 days [26]. 

In a Finnish follow-up study, 47 NE patients were followed for 4 to 8 years after the acute phase [20]. Health-related quality of life assessment was evaluated with the 15D instrument [27]. The results were comparable to that in an age-standardized general reference population [20]. 

In a recent study among 915 NE patients in Northern Sweden, patients answered a questionnaire on demographic and health-related factors, including the Fatigue Severity Scale (FSS). Time to complete recovery was reported to exceed 3 months for 47% and 6 months for 32% of the patients [28]. NE patients had significantly higher FSS scores than the general population group used for comparison. Differences were greater among women than men. Smoking is known to increase the risk and severity of PUUV infection [29,30]. Adjustment for smoking reduced the effect of PUUV infection on the results only slightly in the Swedish study [28]. 

## 7. Fatigue after Other Infections

Long-term fatigue is common after many viral infections. In a study performed in the United Kingdom, this symptom was more common after infectious mononucleosis than after influenza or tonsillitis [31]. Female sex and premorbid mood disorders were risk factors for fatigue.

Post-infectious fatigue was observed in approximately 25% of hospitalized patients with dengue infection in Singapore [32]. A higher frequency of the symptom was observed in older patients and females, but there was no association between the severity of the infection and the development of fatigue after dengue infection [32]. 

Post-discharge symptoms and health-related quality of life after hospitalization for COVID-19 were studied by Garrigues and associates [33] in 120 French patients after a mean of 111 days following admission. The most frequently reported persistent symptoms were fatigue (55%), dyspnea (42%) loss of memory (34%), and concentration and sleep disorders (28% and 31%, respectively) [33]. Fatigue and muscle weakness were also the most common symptoms in a recent Chinese study, in which the median follow-up time of after the onset of COVID-19 infection symptoms was 186 days [34]. 

An Australian prospective cohort study evaluated the risk factors, symptom patterns, and longitudinal course of prolonged illness after EBV -infection (mononucleosis), *Coxiella burnetii*, Q fever, and Ross River virus (epidemic polyarthritis). The post-infectious fatigue phenotype was stereotyped and occurred at a similar incidence after each infection. The symptom was predicted by the severity of the acute illness and it persistent for up to 12 months [35]. 

The pathogenesis of post-infectious fatigue is not known, and it is most probably multifactorial. Host factors, such as age and sex seem to be important in the pathogenesis. Proinflammatory cytokines have an influence on the central nervous system during acute infections, but they do not remain high during post-infectious period [35]. Hormonal deficiencies described above in this review, can cause long-lasting symptoms in some patients. The pathogenesis of chronic fatigue syndrome is outside the scope of this review. 

## 8. Influence of Study Protocols

As mentioned above, follow-up studies performed in Sweden have, thus, shown that PUUV infection is a risk factor for certain cardiovascular diseases and lymphoma. The study population in all these studies consisted of hospital-treated patients. Similar results, however, were not obtained in two seroprevalence studies in Sweden [36,37] and in one in Finland [29]. No associations between PUUV -seropositivity and chronic lung disease, diabetes, hypertension, renal dysfunction, stroke, AMI, and cancer were observed [29,37]. 

We know that the most severe cases of NE are treated at hospital and in serological studies also patients with clinically mild or even totally asymptomatic patients are included. We, however, have no solid data to address the question whether the severity of the acute illness has an influence on long-term consequences of PUUV infection. It is obvious that more studies are needed on this topic. 

## 9. Immune Response 

According to our previous studies, human host-genetics has a clear influence on the clinical severity of PUUV infection. HLA B8-DR3 associates with a severe clinical [38,39] and radiological [40] findings, while HLA B27 [41] with a benign course of the infection. Interestingly, HLA B8-DR3 is known to associate with chronic autoimmune diseases including celiac disease and HLA B27 with polyarthritis and ancylosing spondylitis after certain bacterial infections. These genetic factors or the severity of acute infection, however, did not have any association with the level of blood pressure, renal function, or the amount of proteinuria evaluated six years after NE [42]. 

In addition to PUUV infection, a person can be infected by additional rodent-borne viruses at the same time. We have found a recent Ljungan virus infection in 15/116 patients hospitalized due to acute NE, while lymphocytic choriomeningitis and orthopoxvirus seroconversions were found in five patients and in one of these 116 patients, respectively [43]. No multiple seroconversions in the same patient were detected. It is not known if the concurrent other infections influence the clinical picture or long-term consequences of NE. 

Reactivation of latent EBV is a well-known phenomenon, and this can happen also during acute PUUV infection [44]. It is also interesting that tetanus- and pertussis -specific IgG concentrations were found to be elevated during acute NE, and the increases in tetanus-specific IgG persisted for a year after the infection [45]. These results suggest that persistence of immune memory is facilitated by heterologous boosting of old memory during memory formation against newly encountered antigens. It is possible that these findings are associated with long-lasting symptoms of the patients with PUUV infection. 

## 10. Concluding Remarks

Most patients recover totally from NE caused by PUUV. A total recovery, however, may happen only after several weeks or months after the acute infection. This should be explained to the patients by the physician treating them. In the case of long-lasting symptoms, the hormonal status of the patient should be evaluated by performing a careful anamnestic and clinical examination of the patient and by using targeted laboratory examinations. The existence of possible long-term cardiovascular and renal complications needs further studies using large patient cohorts and long follow-up times. 

## Figures and Tables

**Table 1:** Severe complications of acute Puumala virus infection.

Complication
*Neurological*
Meningoencephalitis
Generalized seizure
Pituitary hemorrhage
Guillain-Barré syndrome
*Cardiopulmonary*
Hypotension and shock
Perimyocarditis
Pulmonary infiltrations and edema
*Hematological*
Disseminated intravascular coagulopathy (DIC)
Multiple bleedings
*Gastrointestinal*
Pancreatitis
Cholecystitis
Splenic rupture
*Others*
Multiorgan failure
Lethal outcome

**Table 2:** Long-term consequences of Puumala virus infection.

Complication
*Cardiovascular*
Acute myocardial infarction
Stroke
Venous thromboembolism
Hypertension
*Nephrological*
Depressed tubular function
Glomerular hyperfiltration
Post-infectious glomerulonephritis
*Endocrinological*
Hypopituitarism
Primary hypothyroidism
Testicular failure
*Others*
Lymphoma
Long-lasting fatigue

## Data Availability

Not applicable.
